# TBHQ Alleviates Particulate Matter-Induced Pyroptosis in Human Nasal Epithelial Cells

**DOI:** 10.3390/toxics12060407

**Published:** 2024-06-03

**Authors:** Ji-Sun Kim, Hyunsu Choi, Jeong-Min Oh, Sung Won Kim, Soo Whan Kim, Byung Guk Kim, Jin Hee Cho, Joohyung Lee, Dong Chang Lee

**Affiliations:** 1 Department of Otorhinolaryngology Head and Neck Surgery, College of Medicine, The Catholic University of Korea, Seoul 06591, Republic of Korea; skswltjs23@catholic.ac.kr (J.-S.K.); kswent@catholic.ac.kr (S.W.K.); kshent@catholic.ac.kr (S.W.K.); coolkim@catholic.ac.kr (B.G.K.); entcho@catholic.ac.kr (J.H.C.); gadori@catholic.ac.kr (J.L.); 2 Clinical Research Institute, Daejeon St. Mary’s Hospital, Daejeon 34943, Republic of Korea; lolo@cmcnu.or.kr (H.C.); yejmdh@cmcnu.or.kr (J.-M.O.)

**Keywords:** fine particulate matter, human nasal epithelial cells, pyroptosis, NLRP3 inflammasome, tert-butylhydroquinone

## Abstract

Pyroptosis represents a type of cell death mechanism notable for its cell membrane disruption and the subsequent release of proinflammatory cytokines. The Nod-like receptor family pyrin domain containing inflammasome 3 (NLRP3) plays a critical role in the pyroptosis mechanism associated with various diseases resulting from particulate matter (PM) exposure. Tert-butylhydroquinone (tBHQ) is a synthetic antioxidant commonly used in a variety of foods and products. The aim of this study is to examine the potential of tBHQ as a therapeutic agent for managing sinonasal diseases induced by PM exposure. The occurrence of NLRP3 inflammasome-dependent pyroptosis in RPMI 2650 cells treated with PM < 4 µm in size was confirmed using Western blot analysis and enzyme-linked immunosorbent assay results for the pyroptosis metabolites IL-1β and IL-18. In addition, the inhibitory effect of tBHQ on PM-induced pyroptosis was confirmed using Western blot and immunofluorescence techniques. The inhibition of tBHQ-mediated pyroptosis was abolished upon nuclear factor erythroid 2-related factor 2 (Nrf2) knockdown, indicating its involvement in the antioxidant mechanism. tBHQ showed potential as a therapeutic agent for sinonasal diseases induced by PM because NLRP3 inflammasome activation was effectively suppressed via the Nrf2 pathway.

## 1. Introduction

The harmful effects of air pollution on human health are well established. Particulate matter (PM) that consists of organic compounds, metals, and dust particles is a significant source of air pollution [1]. Scientific research has demonstrated that exposure to PM can instigate diverse inflammatory responses in several human organs [2]. Categorizing PM based on aerodynamic diameter is a critical criterion, reflecting its capacity for atmospheric mobility and potential for inhalation via the respiratory system. PM particles with a diameter <10 μm pose significant health risks because they can penetrate deep into the alveoli after inhalation through the nasal passages [3]. Evidence shows that PM exposure causes oxidative stress and inflammatory reactions in various human nasal cells, leading to increased research on potential therapeutic interventions [4,5,6].

Research has shown that PM triggers oxidative stress, the generation of pro-inflammatory cytokines, autophagy, and immune cell cytotoxicity mediated by various signaling pathways [7,8,9]. Among the spectrum of inflammatory responses, pyroptosis has recently attracted significant attention due to its strong correlation with the pathophysiology of diseases induced by PM exposure. Pyroptosis is a form of programmed cell death characterized by disruption of the cell membrane, resulting in the release of inflammatory cytokines and triggering subsequent inflammation cascades [10]. In the process, the Nod-like receptor family pyrin domain containing three (NLRP3) inflammasome signaling axis is considered to have a significant function in systemic diseases associated with PM exposure, such as cardiovascular injury, lung inflammation, and corneal damage [11,12,13,14,15].

Globally, synthetic antioxidants are the most widely utilized substances to prevent degradation and extend the shelf life of food, pharmaceuticals, and commercial goods. Among these, tert-butylhydroquinone (tBHQ) is a synthetic antioxidant and antimicrobial agent utilized within allowed limits in numerous foods and products [16]. The antioxidant properties of tBHQ were proposed in several studies to potentially exert therapeutic effects for chronic diseases when administered at specific doses [17,18]. However, whether tBHQ can effectively protect human nasal epithelial cells from oxidative damage induced by PM remains inconclusive, and the underlying mechanisms of the potential protective effects of tBHQ have not been fully elucidated.

This study is designed (1) to determine whether PM induces pyroptosis in human nasal epithelial cells, (2) to assess the role of tBHQ in the NLRP3-mediated pyroptosis pathway, and (3) to evaluate the mechanism of action of tBHQ in PM-induced human nasal epithelial cells. The primary focus of this study is to explore the potential of tBHQ as a therapeutic intervention for sinonasal diseases induced by PM exposure.

## 2. Materials and Methods

### 2.1. Reagents

Eagle’s minimum essential medium (EMEM), Hoechst 33342, antibiotic-antimycotic solution, and fetal bovine serum (FBS) solution were purchased from Gibco (Thermo Fisher Scientific, Waltham, MA, USA). MCC950, tBHQ, the PM standard reference material SRM 2786, and propidium iodide (PI) were obtained from Millipore Sigma-Aldrich (St. Louis, MO, USA). Antibodies against glyceraldehyde phosphate dehydrogenase (GAPDH), NLRP3, cleaved caspase-1, heme oxygenase-1 (HO-1), and NAD(P)H:quinone oxidoreductase 1 (NQO1) were obtained from Cell Signaling Technology (Danvers, MA, USA). Antibodies against lamin B1 and gasdermin D (GSDMD)-N were acquired from Abcam (Cambridge, UK). Nuclear factor erythroid 2-related factor 2 (Nrf2)-targeted small interfering RNAs (siRNAs) as well as transfection reagents and kits were purchased from Santa Cruz Biotechnology (Santa Cruz, CA, USA).

### 2.2. Cell Culture and Treatment

Human nasal epithelial RPMI 2650 cells (ATCC, Manassas, VA, USA) were grown in EMEM culture medium supplemented with 10% FBS and a 1% antibiotic-antimycotic solution. The culture medium was replaced every 2–3 days, and the cells were maintained in a humidified incubator. Cells were seeded when they reached a confluence of 70–80% and were used for experiments the next day. In this experiment, a 50 mg/mL stock solution of PM was prepared in PBS. The group treated with PBS alone is referred to as the control (PBS) group throughout this study. For experiments, cells were seeded at a concentration of 1 × 10^4^ cells per well in 96-well plates, 4 × 10^5^ cells per well in 6-well plates, or 8 × 10^5^ cells in 100-mm dishes, and then treated with PM at the indicated concentration.

### 2.3. Determination of Cell Viability

To evaluate cytotoxicity and cell viability, we conducted an MTT (3-(4,5-dimethylthiazol-2-yl)-2,5-diphenyltetrazolium bromide) assay using the EZ-Cytox Cell Viability Assay Kit from DoGen (Seoul, Republic of Korea). The cells were plated in a 96-well plate at 1 × 10^4^ cells per well and treated with various concentrations of PM (0, 25, 50, or 100 μg/mL) or tBHQ (0, 5, 10, 20, or 40 μM) for 24 h. Before exposing the cells to 50 μg/mL PM, the cells were pre-treated with either 10 μM tBHQ for 1 h or 1 μM MCC950 for 2 h. After 24 h, 10 μL of the MTT assay kit reagent was added to each well, and the plate was further incubated for 2 h. Next, a microplate reader (Bio-Rad, Hercules, CA, USA) was used to measure the absorbance at 450 nm.

### 2.4. Pyroptotic Cell Death Assay

To verify the formation of pores in cell membranes, Hoechst 33342 and PI staining were performed. The cells were seeded at a density of 4 × 10^5^ cells per well in a 6-well plate and subjected to treatment with varying concentrations of PM (0, 25, 50, or 100 μg/mL) or tBHQ (0, 5, 10, 20, or 40 μM) for a duration of 24 h. Prior to their exposure to 50 μg/mL PM, the cells underwent pre-treatment with either 1 μM MCC950 for 2 h or 10 μM tBHQ for 1 h. After the specified treatment, the cells were exposed to a combination of Hoechst 33342 and PI for 25 min at 37 °C. Subsequently, the cells were captured using a fluorescence microscope. Cell culture supernatants were obtained for the evaluation of lactate dehydrogenase (LDH) release and quantified using an LDH kit. Briefly, 10 μL of cell culture supernatant was combined with 100 μL of the LDH reaction mixture and incubated at 37 °C for 30 min. Subsequently, absorbance at 450 nm was measured using a microplate reader (Bio-Rad).

### 2.5. Quantitative Real-Time PCR

Cells were cultured at a density of 4 × 10^5^ cells per well in a 6-well plate. After incubating for 24 h, the cells were pretreated with 10 μM tBHQ for 1 h, followed by the addition of 50 μg/mL PM. Subsequently, the cells were incubated for an additional 24 h.

Total RNA was extracted using the Easy-BLUE^TM^ Total RNA Extraction Kit from iNtRON Biotechnology (Sungnam, Republic of Korea). Briefly, 900 μL of TRIzol was added to the cells, followed by the addition of 100 μL of chloroform (CHCl3) and vortexing for 15 s. The mixture was then centrifuged at 13,000 rpm for 10 min at 4 °C, and the upper phase was carefully transferred to a new Eppendorf tube. An equal volume of cold isopropanol was added to the upper phase and mixed by inversion. This mixture was placed in a −20 °C freezer for 30 min to enhance precipitation, and then centrifuged at 13,000 rpm for 10 min at 4 °C. After discarding the supernatant, 500 μL of ice-cold 75% ethanol (prepared with RNase-free water and stored at −20 °C) was added to the pellet, vortexed, and allowed to stand for 10 min to rinse the pellets. Subsequently, another round of centrifugation at 13,000 rpm for 10 min at 4 °C was performed, and the supernatant was discarded. The pellets were air-dried for 10 min, and finally, 20 μL of RNase-free H_2_O was added to elute the RNA. The RNA was quantified using a Nanodrop One spectrophotometer (Thermo Fisher Scientific).

Using 1 μg of RNA, cDNAs were produced with Reverse Transcriptase Premix (Elpis Biotech, Daejeon, Republic of Korea). Real-time PCR was then conducted on an ABI 7500 FAST system (Applied Biosystems, Foster City, CA, USA). GAPDH mRNA expression served as the normalization reference for relative mRNA levels.

The primer sequences used for PCR analysis in this study were as follows: 5′-GAGAGCCCAGTCTTCATTGC-3′ (forward primer) and 5′-TGCTCAATGTCCTGTTGCAT-3′ (reverse primer) for Nrf2; 5′-CGGGCCAGCAACAAAGTG-3′ (forward primer) and 5′-AGTGTAAGGACCCATCGGAGAA-3′ (reverse primer) for HO-1; 5′-GGGCAAGTCCATCCCAACTG-3′ (forward primer) and 5′-GCAAGTCAGGGAAGCCTGGA-3′ (reverse primer) for NQO1; 5′-AGCCACATCGCTCAGACAC-3′ (forward primer) and 5′-GCCCAA TACGACCAAATCC-3′ (reverse primer) for GAPDH.

### 2.6. RNA Interference

Small interfering RNA (siRNA) targeting Nrf2 was employed to silence the Nrf2 gene, facilitated by Lipofectamine^®^ 3000. Cells were seeded at a density of 4 × 10^5^ cells per well in 6-well plates and cultured until they reached a confluence of 70–90%. Subsequently, they were transfected with 50 nmol of siNrf2. Following a 24-h transfection period with either control-siRNA or Nrf2-siRNA, the cells underwent 1-h pretreatment with 10 μM tBHQ. Afterwards, the cells were exposed to 50 μg/mL PM for 24 h. siControl, which consists of a non-targeting scrambled siRNA, was utilized as a negative control.

### 2.7. Western Blotting

Cells, plated at a density of 8 × 10^5^ cells per 100-mm dish, were subjected to treatment. After rinsing twice with PBS, cell lysis was conducted using RIPA lysis buffer from Elpis Biotech (Daejeon, Republic of Korea), with the addition of protease inhibitor cocktail tablets from Roche Diagnostics (Mannheim, Germany). Subsequently, the lysates were centrifuged at 13,000 rpm for 15 min to isolate the protein fraction. The bicinchoninic acid (BCA) technique was used for quantification of protein concentration in the lysates and was facilitated using the BCA Protein Assay Kit from Pierce (Rockford, IL, USA). Equal quantities of protein were loaded onto the gel, separated using electrophoresis, and subsequently transferred onto nitrocellulose membranes from Bio-Rad. After overnight incubation at 4 °C with rabbit primary antibodies specific to NLRP3 (1:500), caspase-1 (P20, 1:500), GSDMD-N (1:500), Nrf2 (1:500), HO-1 (1:1000), NQO1 (1:1000), lamin B1 (1:500), and GAPDH (1:1000), the blots were incubated with goat anti-rabbit IgG-HRP-labeled secondary antibody for 2 h at room temperature. Following this, bands were visualized using an ECL reaction and identified with a ChemiDocTM XRS+ system from Bio-Rad.

### 2.8. Immunofluorescent Staining

Cells were seeded at a density of 4 × 10^5^ cells per well in 6-well plates and incubated overnight. After the designated treatment, the cells were fixed with 4% paraformaldehyde for 15 min. Subsequently, permeabilization was carried out by exposing the samples to 0.2% Triton-X-100 for 15 min at room temperature. Then, cells were subjected to blocking with 5% bovine serum albumin for 1 h. Next, the primary antibody targeting caspase-1 (P20) was added at a dilution of 1:200, and cells were incubated overnight at 4 °C. After washing, cells were exposed to a secondary antibody, diluted at a 1:1000 ratio, and incubated in the dark for 1 h at room temperature. DAPI staining was then performed for 10 min to visualize the cell nuclei. The stained cells were observed under a fluorescence microscope (Olympus IX73 model; Olympus Corporation, Tokyo, Japan). ImageJ software Version 1.54i (National Institute of Health) was used for quantitative analysis of immunofluorescence intensity from the obtained images.

### 2.9. Oxidative Stress Assay

The cells were cultured at a density of 4 × 10^5^ cells per well in a 6-well plate. After incubating for 24 h, the cells were pre-treated with 10 μM tBHQ for 1 h, and then 50 μg/mL of PM was added. Subsequently, the cells were further incubated for an additional 24 h. The treated cells were collected, cell lysis was performed, and the resulting lysates were subjected to centrifugation to collect the supernatant for subsequent analysis. The content or activity of malondialdehyde (MDA) and superoxide dismutase (SOD) were assessed using commercially available kits. The MDA assay kit was used to measure the level of MDA, a marker of lipid peroxidation, and the SOD assay kit was used to determine the activity of SOD, an antioxidant enzyme.

### 2.10. Supernatant ELISA Detection

The cells underwent pretreatment with 10 μM tBHQ for a duration of 1 h. Following this pre-treatment, 50 μg/mL of PM was introduced to the cells. Subsequently, the cells were allowed to incubate for an additional 24 h. After this specific treatment regimen, the culture medium was collected and then subjected to centrifugation at 1500 rpm for a duration of 5 min to remove any suspended cells. The resulting supernatants were carefully preserved at −80 °C until they could be further analyzed. To assess the protein concentrations of human interleukin (IL)-1β and IL-18 in the culture supernatant, we employed enzyme-linked immunosorbent assay (ELISA) kits specifically designed for these two cytokines. The levels of IL-1β and IL-18 in the samples were quantified by comparing the absorbance values to a standard curve that had been constructed using known concentrations of recombinant cytokines.

### 2.11. Statistical Analysis

GraphPad Prism 5 (GraphPad Software Prism 5 Version 5.03, Inc., San Diego, CA, USA) was used to draw graphs and for statistical analysis. Depending on the experimental design, the Student’s *t*-test or one-way ANOVA and Tukey’s test were carried out. All data were presented as the mean ± standard error of the mean (SEM) of at least three independent experiments.

## 3. Results

### 3.1. PM-Induced Pyroptosis in Human Nasal Epithelial Cells

**Figure 1:**
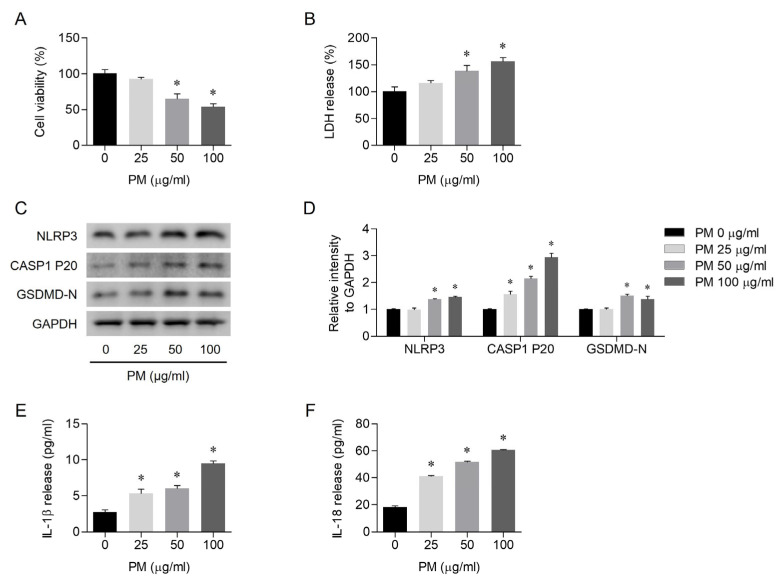
Exposure to particulate matter (PM)-induced pyroptosis in human nasal epithelial cells. (**A**) Treatment with PM at concentrations of 0, 25, 50, and 100 ug/mL resulted in decreased cell viability. (**B**) PM exposure at concentrations of 0, 25, 50, and 100 ug/mL induced cell death, as assessed by the LDH assay. (**C**) PM exposure led to increased expression levels of Nod-like receptor family pyrin domain containing three (NLRP3), cleaved caspase-1, and gasdermin D (GSDMD)-N, as determined by Western blot. (**D**) The relative intensity of protein levels compared with the glyceraldehyde phosphate dehydrogenase (GAPDH) control was determined based on the quantification of the Western blot signals. (**E**,**F**) PM exposure resulted in the release of interleukin (IL)-1β and IL-18, as detected by enzyme-linked immunosorbent assay (ELISA). For all experiments, the data are shown as the mean ± standard error of the mean (SEM). * *p* < 0.05 compared with the control (PBS).

To assess the effects of PM on human nasal epithelial cell viability and the underlying mechanism of pyroptosis, an MTT assay and Western blotting were performed. The cells were treated with different concentrations of PM (0, 25, 50, or 100 μg/mL) for 24 h. After treatment, cell viability decreased in a dose-dependent manner with PM exposure (Figure 1A). PM exposure led to cell death in a dose-dependent manner (Figure 1B). Based on Western blot analysis, PM exposure resulted in the activation of the NLRP3 inflammasome (Figure 1C). The relative intensity of NLRP3, cleaved caspase-1, and GSDMD-N protein levels normalized to the GAPDH control also exhibited an increasing trend proportional to the concentration of PM exposure (Figure 1D). On ELISA, PM exposure resulted in a dose-dependent increase in IL-1β and IL-18 levels, the products of pyroptosis (Figure 1E,F).

### 3.2. PM-Induced NLRP3 Inflammasome-Dependent Pyroptosis

**Figure 2:**
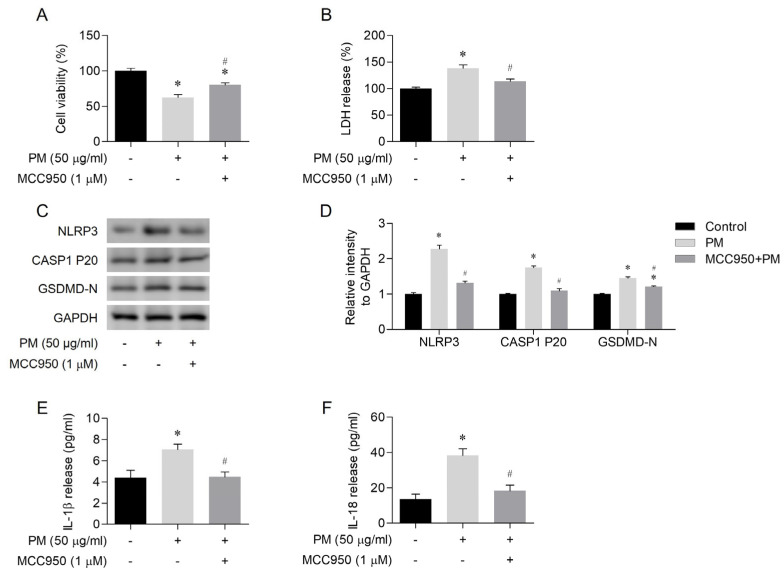
Particulate matter (PM)-triggered pyroptosis of human nasal epithelial cells in a Nod-like receptor family pyrin domain containing three (NLRP3) inflammasome-dependent manner. (**A**) MCC950 (NLRP3 inflammasome inhibitor) increased cell viability compared to the PM-only-treated group. (**B**) MCC950 decreased cell death compared to the PM-only-treated group. (**C**,**D**) MCC950 decreased the expression levels of NLRP3, cleaved caspase-1, and gasdermin D (GSDMD)-N as assessed by Western blot. (**E**,**F**) MCC950 caused a decrease in the release of IL-1β and IL-18 as measured by ELISA. For all experiments, the data are shown as the mean ± SEM. * *p* < 0.05 indicates a difference from the control (PBS), while ^#^ *p* < 0.05 represents a difference compared with the PM-only-treated group. The symbols “+” and “−” in the figure indicate the presence and absence of the respective treatments.

The effects of MCC950, a specific inhibitor targeting the NLRP3 inflammasome, were investigated in human nasal epithelial cells exposed to PM. The cells were pretreated with 1 μM MCC950 for 2 h before incubating with PM (50 μg/mL) for an additional 24 h. In the cells pretreated with MCC950, PM-induced cell death was attenuated (Figure 2A,B). Western blot analysis showed a reduced expression of NLRP3, cleaved caspase-1, and GSDMD-N in the MCC950-treated cells (Figure 2C,D). IL-1β and IL-18 levels were decreased in the MCC950-treated cells compared with the PM-only-treated cells (Figure 2E,F). Based on the findings, PM-induced pyroptosis was likely mediated by the NLRP3 inflammasome.

### 3.3. Effects of tBHQ on PM-Induced Pyroptosis

**Figure 3:**
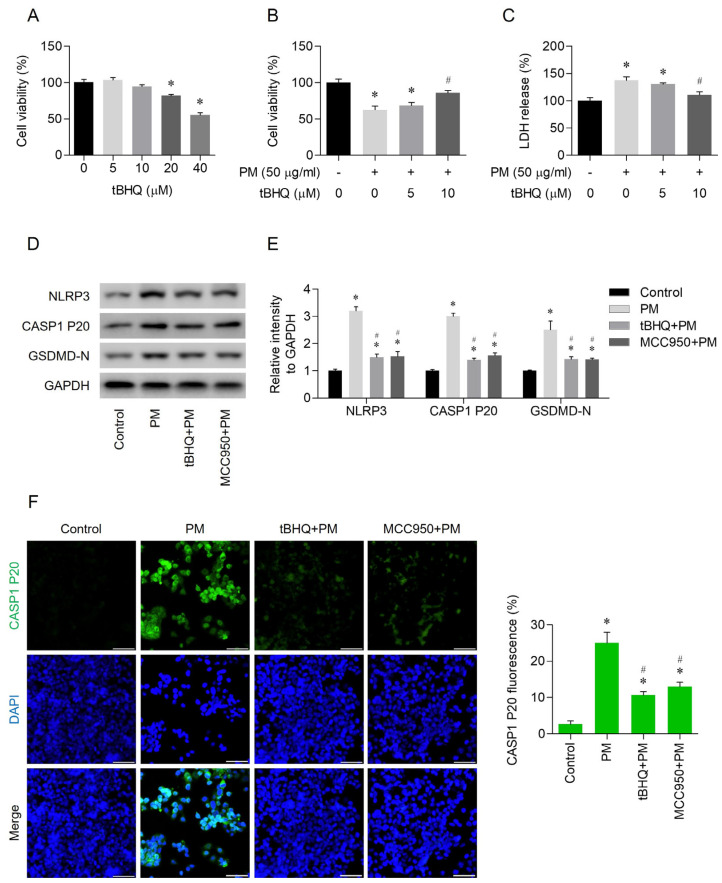

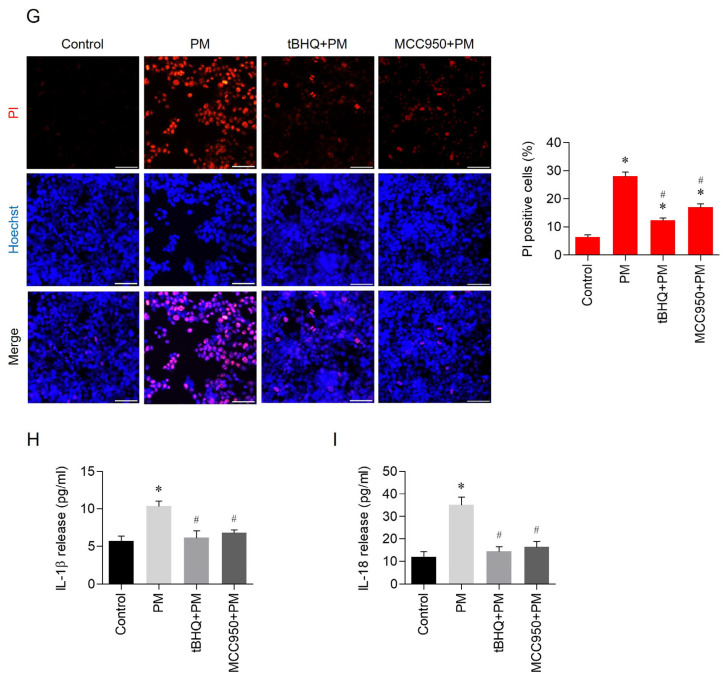
tBHQ suppressed PM-induced NLRP3 inflammasome-mediated pyroptosis in human nasal epithelial cells. (**A**) The cytotoxicity of tBHQ was evaluated using the MTT assay. (**B**) tBHQ demonstrated a protective effect against PM-induced cytotoxicity. (**C**) tBHQ reduced cell death as assessed by the LDH assay. (**D**,**E**) tBHQ downregulated the expression levels of NLRP3, cleaved caspase-1, and GSDMD-N, as determined by Western blot. (**F**) tBHQ attenuated the expression of activated caspase 1. (**G**) tBHQ preserved cell viability against PM-induced cell death, as indicated by PI staining. PI was stained red and DAPI was stained blue. (**H**,**I**) tBHQ attenuated the release of IL-1β and IL-18, as measured by ELISA. For all experiments, the data are shown as the mean ± SEM. * *p* < 0.05 indicates a difference from the control (PBS), and ^#^ *p* < 0.05 represents a difference compared with the PM-only-treated group. Scale bar = 100 μm.

Cells were treated with various concentrations of tBHQ (0, 5, 10, 20, or 40 μM) for 24 h. The tBHQ concentrations that did not affect cell viability were identified using the MTT assay (Figure 3A). In addition, the cells were pretreated with 5 μM or 10 μM tBHQ for 1 h. After pre-incubation, the cells were exposed to 50 μg/mL PM and then incubated for an additional 24 h. tBHQ attenuated PM-induced cell death (Figure 3B,C) and, similar to the selective NLRP3 inhibitor MCC950, decreased the activity of NLRP3, cleaved caspase-1, and GSDMD-N (Figure 3D,E). Based on immunofluorescent staining, both tBHQ and MCC950 reduced the activity of CASP1 P20, the active subunit of caspase-1 (Figure 3F). Hoechst and PI staining, methods used to identify pyroptotic cell death, confirmed that tBHQ and MCC950 inhibited pyroptosis (Figure 3G). Both tBHQ and MCC950 led to a reduction in IL-1β and IL-18, cytokine products of pyroptosis (Figure 3H,I).

### 3.4. The Antioxidant Effects of tBHQ Via the Nrf2/HO-1 Pathway in PM-Treated Human Nasal Epithelial Cells

**Figure 4:**
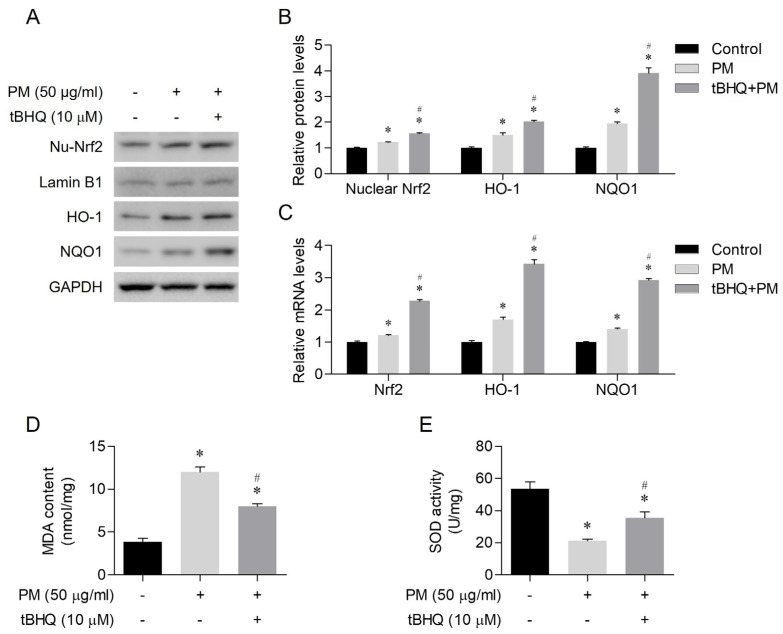
tBHQ activated the Nrf2/HO-1 pathway and attenuated oxidative stress in human nasal epithelial cells treated with PM. (**A**,**B**) tBHQ induced the activation of nuclear Nrf2 along with elevated protein expression levels of HO-1 and NQO1. (**C**) tBHQ upregulated the mRNA levels of Nrf2, HO-1, and NQO1. (**D**) tBHQ mitigated the increase in PM-induced MDA levels. (**E**) tBHQ increased SOD enzyme activity. * *p* < 0.05 indicates a difference from the control (PBS), and ^#^ *p* < 0.05 represents.

Prior to the application of 50 μg/mL PM, the cells were pretreated with 10 μM tBHQ for 1 h. Subsequently, the cells were incubated for an additional 24 h. tBHQ led to the activation of nuclear Nrf2 and increased protein expression levels of HO-1 and NQO1 (Figure 4A,B). In addition, tBHQ induced upregulation of Nrf2, HO-1, and NQO1 mRNA levels (Figure 4C). tBHQ reduced the levels of MDA, a product of oxidative stress, and increased expression of the antioxidant enzyme SOD (Figure 4D,E). Thus, tBHQ was considered to exert antioxidant actions via the Nrf2/HO-1 pathway.

### 3.5. Nrf2 Knockdown Abolished the Pyroptosis-Inhibiting Effects of tBHQ

**Figure 5:**
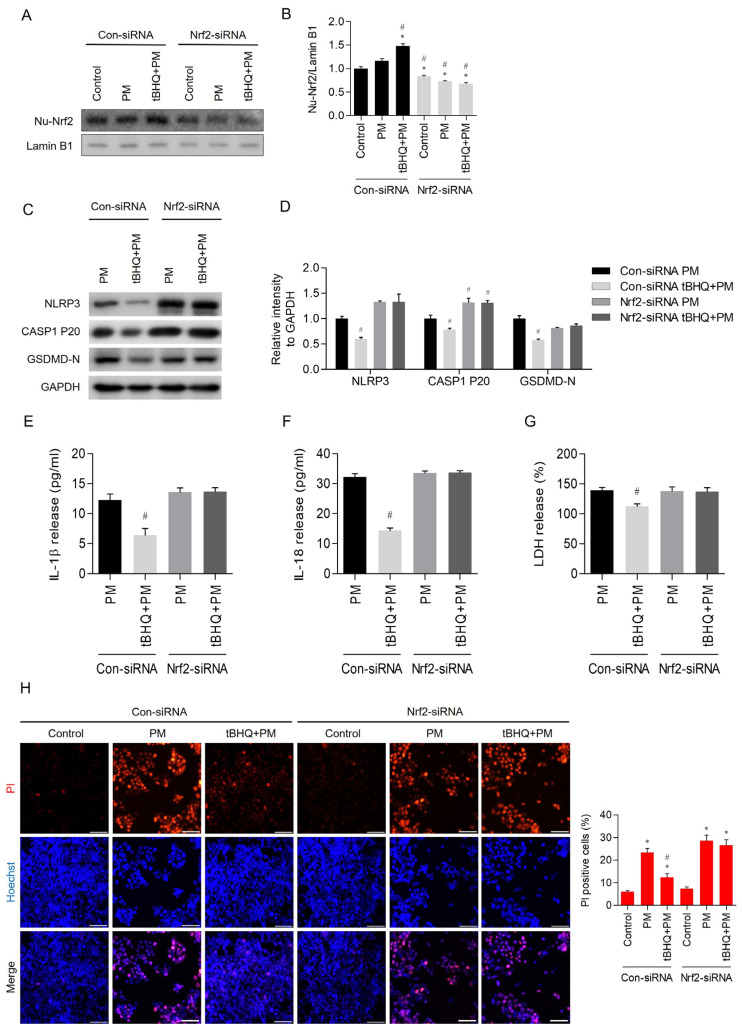
Knockdown of Nrf2 using Nrf2-siRNA abolished the protective effect of tBHQ on NLRP3-mediated pyroptosis induced by PM. (**A**,**B**) Knockdown of Nrf2 using Nrf2-siRNA abolished the ability of tBHQ to reduce the expression levels of nuclear Nrf2. (**C**,**D**) Knockdown of Nrf2 using Nrf2-siRNA abrogated the ability of tBHQ to reduce the expression levels of NLRP3 inflammasome-related proteins, including NLRP3, caspase-1, and GSDMD-N. (**E**,**F**) Knockdown of Nrf2 using Nrf2-siRNA abrogated PM-induced release of IL-1β and IL-18. (**G**) Knockdown of Nrf2 by Nrf2-siRNA abolished the ability of tBHQ to reduce PM-induced LDH release. (**H**) Knockdown of Nrf2 using Nrf2-siRNA abolished the efficacy of tBHQ in reducing PM-induced propidium iodide positivity. PI was stained red and DAPI was stained blue. For all experiments, the data are shown as the mean ± SEM. * *p* < 0.05 indicates a difference from the control (PBS), and ^#^ *p* < 0.05 represents a difference compared with the PM-only-treated group. Scale bar = 100 μm.

Following 24-h transfection with either control-siRNA or Nrf2-siRNA, cells were pre-treated with 10 μM tBHQ for 1 h and subsequently exposed to 50 μg/mL PM for 24 h. Knockdown of Nrf2 using Nrf2-siRNA significantly abolished the ability of tBHQ to reduce the expression levels of nuclear Nrf2 (Figure 5A,B). In the absence of functional Nrf2, the tBHQ-mediated reduction in NLRP3 inflammasome-related proteins, including NLRP3, cleaved caspase-1, and GSDMD-N, was significantly reduced (Figure 5C,D). Further, ELISA results indicated that the tBHQ-induced decrease in the levels of IL-1β and IL-18 was abolished by knockdown of Nrf2 (Figure 5E,F). The protective effect of tBHQ against PM-induced pyroptosis was also eliminated in Nrf2-siRNA-transfected cells, as evidenced by the increased LDH release (Figure 5G) and higher staining of dead cells with propidium iodide observed through a fluorescence microscope (Figure 5H).

## 4. Discussion

PM consists of a mix of solid, liquid, or hybrid particles in the air with complex compositions [19]. The impact of PM is influenced by individual breathing patterns, but particle size is crucial for its health effects [20]. Smaller particles penetrate deeper into the respiratory tract, with those under 10 μm posing significant health risks [1,21]. Prolonged PM exposure can irritate the respiratory mucosa and trigger or worsen inflammation, disrupting nasal epithelial barriers and causing mucosal swelling [22]. This exacerbates conditions like allergic rhinitis and rhinosinusitis, with studies linking PM levels to chronic rhinosinusitis severity [23,24]. Mice exposed to PM show inflammatory cell infiltration and increased cytokine levels, along with airway barrier disruption [25]. Research has increasingly focused on how PM triggers inflammatory responses at the cellular level through complex signaling pathways. Clarifying the mechanisms by which PM impacts nasal epithelial cells and identifying the regulatory factors of these inflammatory responses is essential for understanding nasal diseases, the first point of PM contact.

In this study, we verified that PM induces pyroptosis in human nasal epithelial cells. When human nasal epithelial cells were co-treated with PM and MCC950, a selective NLRP3 inflammasome inhibitor, we observed a reduction in pyroptosis. Through this, it was determined that pyroptosis likely proceeds in an NLRP3 inflammasome-dependent manner. Pyroptosis induces pathological ion fluxes that lead to cellular lysis and the release of inflammatory intracellular contents [26]. This phenomenon is primarily controlled by caspase-1 and associated with activation of the NLRP3 inflammasome [10]. Inflammasomes are clusters of proteins found in the cytoplasm, and they play a crucial role in regulating the immune response when the body is exposed to microbial infections or experiences cellular damage [27]. The assembly of an inflammasome leads to the enzymatic activation of procaspase-1, resulting in the active caspase-1 [28]. Active caspase-1 plays a role in cleaving precursor forms of inflammatory cytokines like IL-1β and IL-18, as well as other proteins, including GSDMD, into their active mature peptides [29]. Among various types of inflammasomes, the NLRP3 inflammasome has been the subject of extensive research, given its potential implication in various human diseases [30]. NLRP3 serves as an intracellular receptor that detects various stimuli, initiating the assembly and activation of NLRP3 inflammasomes [31]. In recent studies, PM reportedly induced NLRP3 inflammasome-dependent pyroptosis in various organ cells, including the eyes, brain, and lungs [14,32,33]. In this study, we observed that PM induces pyroptosis in nasal epithelial cells, and the mechanism was dependent on the NLRP3 inflammasome. Clinically, the NLRP3 inflammasome could be a crucial therapeutic target for nasal diseases caused by PM exposure.

We confirmed that tBHQ inhibits the NLRP3 inflammasome, thereby alleviating pyroptosis. Additionally, tBHQ protects against PM-induced pyroptosis in nasal epithelial cells through the Nrf2/HO-1 pathway’s antioxidant activity. Synthetic phenolic antioxidants like tBHQ interact with peroxides in food, preventing deterioration and enhancing stability, thus extending shelf life [16,34]. tBHQ stabilizes products such as vegetable oils and margarine, resulting in consistent human exposure through processed food consumption. While high doses of tBHQ have been linked to adverse effects in animals, including gastrointestinal tumors and DNA damage [35], other studies highlight its therapeutic antioxidant effects. tBHQ upregulates antioxidant genes, preventing necrotic cell death in human neural stem cells [36] and exhibiting hepatoprotective effects through autophagy in hepatocytes [37]. These benefits primarily involve Nrf2 activation, as substantial evidence shows tBHQ as a significant Nrf2 activator [36,38].

Nrf2 is a nuclear protein known for its role in antioxidant responses and cellular detoxification [39]. Activated by oxidative stress or chemical stimuli, Nrf2 moves to the nucleus and promotes the expression of about 500 genes involved in redox balance, detoxification, stress response, and metabolism [40,41]. It is key to protecting cells from oxidative stress and chemical toxicity in various conditions, including inflammation, neurological disorders, and cancer [42,43]. In our study, knockdown of Nrf2 using Nrf2-siRNA in PM-treated epithelial cells abolished the inhibitory effect of tBHQ on the pyroptosis-related NLRP3 pathway (Figure 5C,D). This suggests that the Nrf2 pathway plays a critical role in the mechanism by which tBHQ inhibits pyroptosis. Although the full interaction between pyroptosis and antioxidant responses remains unclear, our findings link pyroptosis to antioxidant reactions in PM and tBHQ-treated nasal epithelial cells. Previously, a close interaction was reported between NLRP3 and reactive oxygen species (ROS). ROS has a significant effect on the initial stages of NLRP3 inflammasome activation, directly influencing the NLRP3 protein and triggering the activation of the NLRP3 inflammasome [44]. The antioxidant action that reduces ROS levels can inhibit the NLRP3 inflammasome and suppress pyroptosis, which has led to growing interest in the therapeutic potential of Nrf2-activating antioxidant compounds for various diseases [45,46,47]. These antioxidant compounds are presumed to inhibit inflammatory responses such as immune cell activation and infiltration caused by ROS and to be involved in innate immune responses associated with the activation of the NLRP3 inflammasome, which was confirmed based on the results of the present study [44]. Both pyroptosis and antioxidant responses are cellular mechanisms that respond to oxidative stress, and specific interactions may exist between these two processes.

The study has certain limitations, including its confinement to a single cell line and the absence of primary cell culture or animal experiments, which results in a dataset with limited depth. Moreover, the intricacies of redox perturbations and their effects on the activation process of the NLRP3 inflammasome are not fully understood. Further investigations are necessary to elucidate the in vivo interactions. The subsequent results will increase understanding regarding the pathophysiological mechanisms of inflammatory diseases associated with PM exposure, potentially leading to the discovery of effective therapeutic agents.

## 5. Conclusions

In human nasal epithelial cells, PM-induced pyroptosis occurs via the NLRP3 inflammasome. tBHQ might have potential as a therapeutic agent for PM-induced sinonasal diseases due to its ability to regulate ROS levels and inhibit NLRP3 inflammasome activation through the Nrf2 pathway. A deeper understanding of the interactions between NLRP3 inflammasome-dependent pyroptosis and antioxidant mechanisms is needed. This knowledge may contribute to the development of potential therapeutic agents for various inflammatory diseases.

## Data Availability

The data presented in this study are available on request from the corresponding author.
